# A Comparison Between Single- and Multi-Scale Approaches for Classification of Histopathology Images

**DOI:** 10.3389/fpubh.2022.892658

**Published:** 2022-07-04

**Authors:** Marina D'Amato, Przemysław Szostak, Benjamin Torben-Nielsen

**Affiliations:** ^1^Roche Information Solutions, F. Hoffmann-La Roche AG, Basel, Switzerland; ^2^Personalized Healthcare, Billennium by Order of Roche Polska Sp. z o.o., Warsaw, Poland

**Keywords:** digital pathology, deep learning, multi-scale analysis, multi-label classification, multiple instance learning, representation learning

## Abstract

Whole slide images (WSIs) are digitized histopathology images. WSIs are stored in a pyramidal data structure that contains the same images at multiple magnification levels. In digital pathology, most algorithmic approaches to analyze WSIs use a single magnification level. However, images at different magnification levels may reveal relevant and distinct properties in the image, such as global context or detailed spatial arrangement. Given their high resolution, WSIs cannot be processed as a whole and are broken down into smaller pieces called tiles. Then, a prediction at the tile-level is made for each tile in the larger image. As many classification problems require a prediction at a slide-level, there exist common strategies to integrate the tile-level insights into a slide-level prediction. We explore two approaches to tackle this problem, namely a multiple instance learning framework and a representation learning algorithm (the so-called “barcode approach”) based on clustering. In this work, we apply both approaches in a single- and multi-scale setting and compare the results in a multi-label histopathology classification task to show the promises and pitfalls of multi-scale analysis. Our work shows a consistent improvement in performance of the multi-scale models over single-scale ones. Using multiple instance learning and the barcode approach we achieved a 0.06 and 0.06 improvement in F1 score, respectively, highlighting the importance of combining multiple scales to integrate contextual and detailed information.

## Introduction

During the past decade, deep learning techniques have shown great potential in the analysis of digitized histopathology images. Digitized histopathology images are generally referred to as “whole-slide images” (WSIs). WSIs are gigapixel-sized images that provide important information for diagnosing diseases and, given their huge dimensions, analyzing them by hand is expensive and time-consuming.

The high resolution of WSIs prevents us from processing these images as a whole and therefore WSIs are broken down into smaller pieces called tiles. Tile-based analysis is useful for tasks where the location of a specific pattern in the image is important. On the other hand, in many histopathology classification tasks, the analysis needs to be performed at the slide-level, that is, whether the entire WSI contains a specific pattern or not. For instance, does a WSI contain a tumor?

When performing a slide-level analysis, it is important to aggregate the tile-level predictions into a single-prediction for the whole image. Typical solutions include a type of weakly supervised learning called multiple instance learning (MIL). MIL is a framework where each WSI is considered as a bag of instances (the tiles) and only the bag label (e.g., the slide label) is known and assigned to all instances contained in the bag (1). Typically, MIL approaches include operators such as mean or max pooling, but the use of attention networks has also become widespread (2). An alternative approach is based on representation learning that consists in obtaining a lower-dimensional representation of the WSI (3). A recent study has shown the promise of representation learning introducing a new method based on clustering tile-level embeddings (4). We refer to this approach as the “barcode approach”.

WSIs are characterized by a pyramidal structure consisting of the same image stored at different spatial resolutions. Most deep learning models in digital pathology require an a-priori choice of one specific magnification at which to perform the analysis. These models do not take advantage of the multi-scale nature of this type of data. Different magnification levels are usually required to recognize different features at a macroscopic scale such as the organ to which the image belongs and at a microscopic scale such as tumor-related information. Pathologists generally conduct their analysis under a microscope combining different scales: they start looking at the tissue at low magnification levels for macroscopic features and then they zoom in into the region of interest to examine the microscopic features at high magnification (5). The pathologists' approach highlights the importance of combining macroscopic and microscopic information obtained from different scales. Although most of the studies in digital pathology use one fixed magnification level, some approaches to integrate multiple magnification levels have recently been explored.

**Figure 1:**
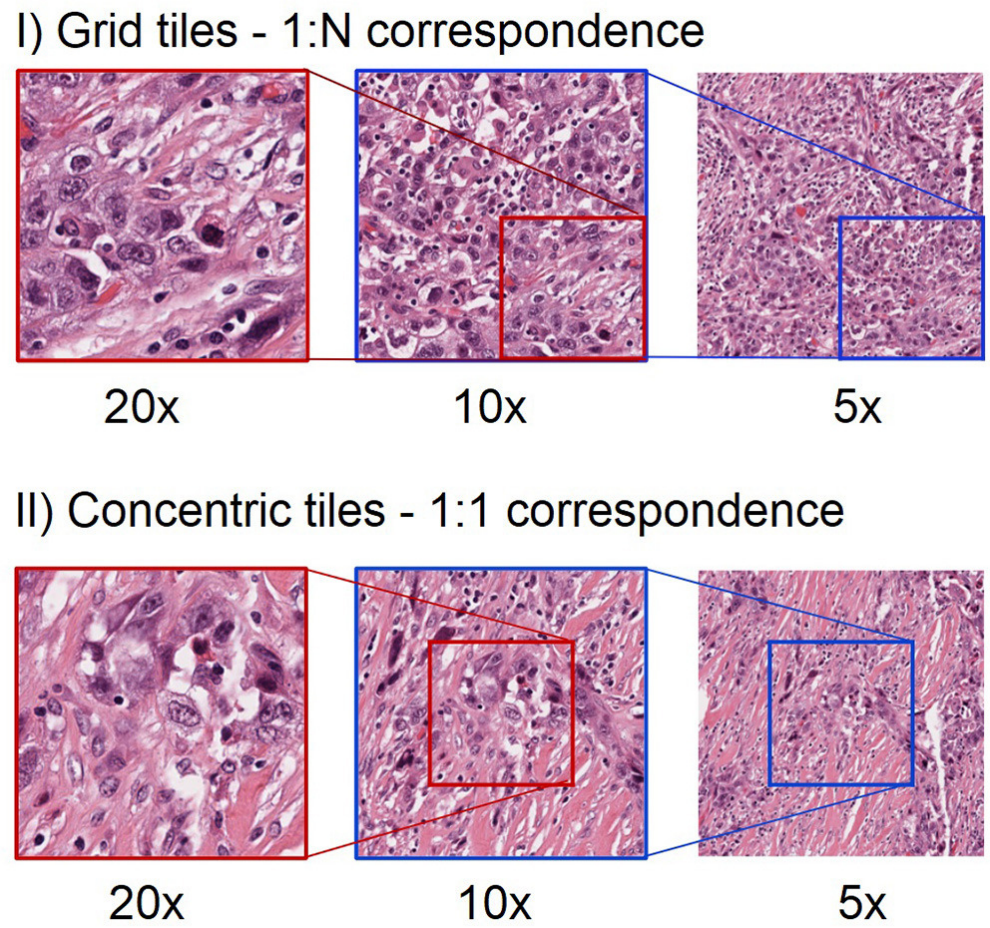
Different approach to extract tiles at multiple magnification levels. The first row shows an example of grid multi-scale tiles. Each tile at 5x can be linked with 4 tiles at 10x and 16 tiles at 20x. Hence, there is a 1:N mapping between tiles from the highest magnification level to the next lower magnification level. Note that for clarity we only show one such tile in this illustration. The second row shows an example of concentric tiles. In this case, each tile at 20x is considered as the centroid for the tiles at lower magnification levels, resulting in a 1:1 correspondence between tiles at different magnifications. When using concentric tiles there will be large overlaps between tiles at lower magnification levels. When using grid tiles, there is no such overlap.

Chiefly, there are two approaches to incorporate multi-scale information from WSIs (Figure 1) (6). First, the concentric approach consists of using tiles centered on the same location of the whole-slide image, with the same size and different magnifications. Different studies have used this approach both for segmentation (7, 8) and for classification (9, 10) tasks. Second, the grid approach starts by splitting the WSIs into a grid of tiles for each magnification level. The tiles that come from the same region are concatenated subsequently. In the grid approach, the number of tiles at different magnifications is different and each tile at lower magnification can be linked with multiple tiles at higher magnifications (11, 12).

In this work, we show a complete pipeline from tile extraction to training of architectures with a multi-scale variant, showing the benefits and challenges of extracting and combining tiles at multiple resolutions. We present a novel approach to expand the barcode method to work with multiscale images. We provide an in-depth comparison between single-scale and multi-scale models to address a multilabel classification problem in digital pathology. To illustrate generalizability, we exploit different training methods to corroborate our hypothesis and we identify promises and pitfalls of multi-scale approaches for the classification of histopathology images. Our findings demonstrate the importance of analyzing these images at different magnification levels to integrate contextual and detailed information.

The remainder of the paper is organized as follows: first, in the “Methods” section, we describe the dataset, preprocessing pipeline, classification methods used and experiment setup. Then, in the “Results” section, we show comparative results between the single- and multi-scale models. Finally, in the “Conclusion and discussion” section we summarized this work and provide guidelines on when multi-scale approaches are the most beneficial.

## Methods

**Figure 2:**
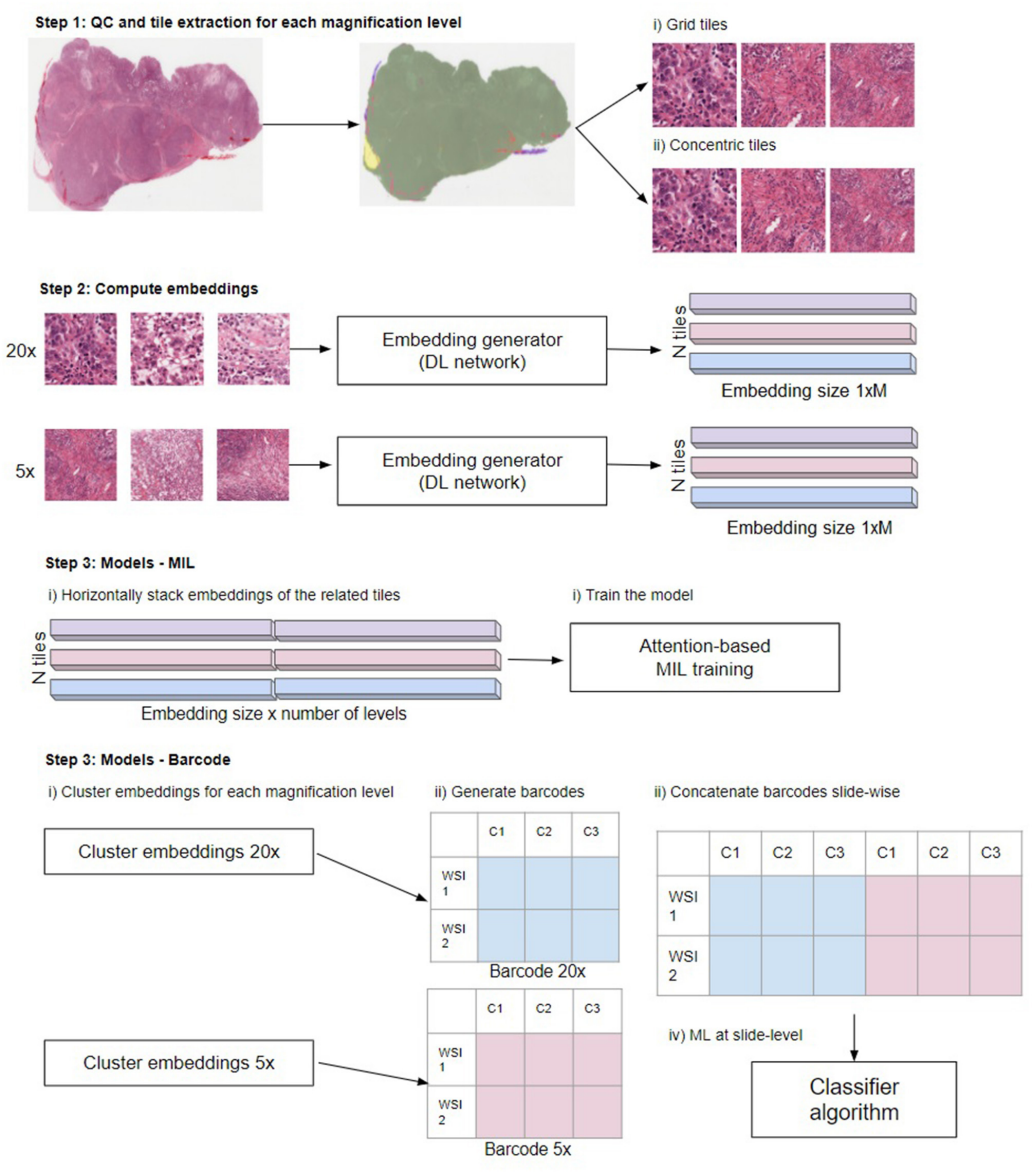
Overview of the steps in the different multi-scale approaches used in this work. Step 1: Perform QC to extract in-focus tiles that contain tissue not covered by pen markers. Extract tiles for each magnification level using the grid and concentric approach. Step 2: Compute the embeddings from the tiles to obtain a 1xM representation for each tile, where M depends on the network (see main text “Compute embeddings”). Step 3, MIL: Train the model. Horizontally stack the embeddings of the related tiles. When using grid tiles, the same embedding at lower magnifications is duplicated and linked with different embeddings at higher magnification. Then, we provide the stacked embeddings as the input to the model. Step 3, barcode method: first cluster the embeddings for each magnification level. Subsequently, generate the barcodes (see reference 4 for details) and slide-wise concatenate the barcodes across magnifications. Finally, the thus created barcodes are used as input to a classifier: Illustration in Step 3 modified from Gueréndel et al. (4).

The problem tackled in this paper is a multilabel classification on a publicly available “The Cancer Genome Atlas” (TCGA) dataset (13). Specifically, we aimed to classify 30 distinct tumor types in histopathology slides. Our methodology is illustrated in Figure 2. First, we ran a quality control algorithm on our WSIs and we extracted the grid and concentric tiles. As a baseline, we used the tiles to train a plain deep learning-based classifier and we aggregated the predictions to generate a slide-level prediction. Then, we computed embeddings from the tiles using both BYOL (14). ResNet self-supervised embeddings and embeddings obtained from a plain ResNet classifier. Finally, based on those embeddings, we assessed single- and multi-scale models using the MIL (2) and barcode approaches.

### Data

All of the proposed experiments were performed on a publicly available dataset obtained from The Cancer Genome Atlas (TCGA). From the complete dataset, only the diagnostic slides fixated via the formaldehyde fixation and paraffin embedding (FFPE) procedure were selected as they are generally of higher quality compared to fast-frozen (FF) slides. Thirty different cancer types were taken into account for this multilabel classification task. When a patient had more than one image (for instance from the bottom and top of the tissue block), we removed the additional ones so that each patient has only one image to avoid overfitting on patient-related properties. The list of the cancer types and their corresponding acronyms can be found in Supplementary Table 1. In total, we used 8,859 WSIs.

Before the extraction, we ran our in-house quality control algorithm for artifact detection to determine the tissue regions and remove adipose tissue, out-of-focus areas or regions covered by pen markers. We extracted 224x224 tiles at three magnification levels (5x, 10x, and 20x) using both the centroid approach and the grid approach. The dataset was partitioned into training and test sets. For more details on the dataset composition and the support of the classes in each set, see Supplementary Table 2.

Data leakage is a major concern for reproducibility of the results. To combat this undesirable behavior the split was performed by stratifying on the cancer type and using the Patient-Wise protocol (15) to avoid any bias caused by having tiles from the same slide in different partitions.

As can be seen from Supplementary Table 2, the dataset is highly unbalanced, which can be a source of a data leakage (15). We tackled this problem by comparing two different methods: (i) we selected all the slides and during the training phase we used a median frequency balancing loss (16) to assign higher weights to the losses of minority classes; (ii) we performed undersampling by randomly sampling 100 slides for each cancer type or the maximum number of WSIs when fewer WSIs were available (which yielded 2,727 slides) and used a cross-entropy loss function. Given that the concentric tiles are heavily storage consuming, the concentric approach experiments were only performed using the undersampling method.

The full TCGA dataset for tiles extracted with a grid method has 5,714,689 tiles for 5x, 22,515,282 for 10x and 92,923,166 for 20x magnification. Average tile size is around 160 KB, which in total makes around 18 TB, out of which the 20x magnification tiles use around 14 TB. Downloading a full dataset using a concentric method would take up around 42 TB.

Training the neural networks responsible for self-supervised embeddings (see below) and classification was done on 8 Nvidia Tesla T4 GPU's; the barcode classifier was trained on a compute unit with 96 CPU cores and 742 GB RAM.

### QC and Tile Extraction

Before the extraction of tiles, we performed an in-house quality control step to locate regions in the WSI that are in-focus and free of adipose tissue or pen markers. We extracted only tiles that contain tissue without artifacts and do not contain adipose tissue (see Figure 2, “Step 1: QC and tile extraction for each magnification level”). The extraction was performed using both the grid approach and the concentric approach (see Figure 1) to train single-scale and multi-scale models. We use in-house cloud software build on top of the OpenSlide (17) library, which provides a straightforward interface to read WSIs and to extract tiles of specific sizes at any magnification levels.

### Compute Embeddings

Both the MIL-approach and the barcode-approach take as input not the tiles themselves but the tile-level embeddings, which is a one dimensional vector representation of a tile image (see Figure 2, “Step 2: Compute embeddings”). They can be obtained from any deep learning network that generates a latent variable. Latent variables are hidden, internal representations of network inputs, learned through the training of the network. Generally, the latent variable is taken from the layer before the classification layers in a deep neural network. We investigated two different approaches to compute the tile embeddings. In the first approach, we used the embeddings generated by our baseline model. That is, the embeddings were generated by a plain vanilla ResNet18 model (18) that was optimized to perform the classification task at hand. The labels were defined weakly as the images are not annotated and some regions of the WSI might not be informative of the response variable. Each tile was assigned the label of the entire WSI, so different tiles from the same slide shared the same label. During validation and test, the tile scores were aggregated at a WSI-level using mean pooling. The embeddings are the results of the layer preceding the fully connected layer; for ResNet18 the embedding vector is 512-dimensional.

Second, we used the self-supervised “Bootstrap your own latent” (BYOL) method (14) to generate embeddings agnostic of the specific task. BYOL is a method that uses contrastive learning in a self-supervised way. Using BYOL, we optimized another ResNet18 model. Again, the embeddings were taken from the last layer before the classification layers resulting in a 512-dimensional embedding. By using the BYOL optimization, we were able to assess if our methods are able to capture meaningful information from the tiles independently of the approach used to generate their embeddings.

We trained a distinct ResNet18 network using the BYOL self-supervised learning paradigm for each magnification level. The training set consisted of all previously extracted TCGA tiles. To ensure high data diversity and to speed up training, we subsampled N tiles from each slide at each epoch (500 tiles for 5x and 10x, 1,000 tiles for 20x magnification). The number of epochs used for training the BYOL network varied so that each network was presented with roughly the same number of input images (e.g., tiles) to make a fair comparison.

### Models

To obtain a slide-level prediction, we used two approaches to aggregate the tile-level embeddings to a slide-level one.

First, we used the embeddings to train an attention-based multiple-instance learning model. In this setting, each WSI is considered as a bag B containing multiple tiles *B* = {*x*_1_, *x*_2_, …, *x*_*n*_}. Each bag has a label y and the tile labels are unknown. The attention mechanism from (2) uses a deep neural network to assign different weights to the embeddings of a bag. The MIL pooling consists of a sum of the embeddings weighted by their attention, then the weighted sum is aggregated and passed through a classifier in order to get a WSI-level prediction. This approach is highly interpretable as key instances (e.g., tiles) in the WSI are assigned higher weights allowing recognition of regions of interest in the images.

We trained our attention MIL model using the embeddings generated from the baseline ResNet18 and the ones from the agnostic BYOL training (as explained in the previous subsection). We trained the MIL algorithm at single-scale levels for all the selected magnification levels and at multi-scale level using the different combinations of magnification levels. To train the classifier on multiple scales, we concatenated the embeddings of the related tiles (see Figure 2, “Step 3—MIL”). For the concentric approach, we have the same number of tiles for all the levels, as there is a one-to-one correspondence between the tiles at different scales. In the grid approach, there are fewer tiles extracted at lower magnification than at higher levels. This means that the embeddings from a lower magnification tile need to be duplicated and concatenated to the embeddings of its higher magnification tiles: for every 10x tile, we obtain four different embeddings for the combined 20x−10x multi-scale (see Figure 1). To reduce overfitting, we performed a hyperparameter tuning that involved the number of instances in each bag, the learning rate, the weight decay and the dropout-rate.

Second, we used a recently proposed representation-learning method from (4). This method allows us to obtain a small but meaningful representation of WSIs using a clustering approach (see Figure 2, “Step 3—Barcode”). After the generation of the tile embeddings, the embeddings of the tiles from the same slide are combined in an NxM representation where N stands for the number of tiles and M is the size of the embedding vector. This matrix is used as input to a clustering algorithm to obtain K clusters for each slide. After that, a barcode is generated for each WSI by calculating the proportion of tiles associated with that WSI that belong to each of the clusters. Finally, the barcodes are used to feed a machine learning classifier to predict slide-level labels. This approach is also highly interpretable as the feature importance of the classifier translates to specific clusters with distinct characteristics, which can be visually inspected by an expert.

In the barcode approach, we use BYOL embeddings for each magnification that were clustered using the Mini Batch K-means (*K* = 150) method. WSIs with <64 tiles in any of the magnifications were discarded. For classification, the XGBoost (19) method was used, with hyperparameters tuned (learning rate, maximum depth, subsample ratio, subsample ratio of columns, regularization) and samples weighted based on class prevalence in the training dataset. To train the classifier on multiple scales, WSI barcodes obtained on each magnification were concatenated slide-wise before classification (see Figure 2, “Step 3—Barcode”).

## Results

The experiments were conducted using a Data Analysis Plan as suggested in Bussola et al. (15), to ensure the highest degree of reproducibility. The dataset was split into training set and test set, consisting of 80 and 20% of the data, respectively. The test set was only used for the final evaluation of the model. Because the dataset is highly imbalanced, we used a macro F1 score as the main metric to compare model performances. The macro F1 is the arithmetic mean of F1 scores for each class. Additionally, we used the weighted F1 score, which is the weighted mean of F1 scores per class, with the weight being the size of the class. Small differences between those metrics indicate that the model performs equally well in classifying classes that have either low or high support. We also employed a robust metric, Matthews Correlation Coefficient (MCC) (20), to confirm the obtained results were free from bias. The classification using barcode approach was performed with 5 fold Monte Carlo cross-validation, calculating 95% confidence interval (CI) (see Supplementary Table 3). Due to operating on very large dataset and high compute power requirements with MIL training, it was not feasible to apply cross-validation to the method.

As a benchmark, we used a state of the art method (21), which utilizes ResNet18 to perform tile-level prediction. During training, we performed standard data augmentation techniques such as rotations, flipping and color augmentation. For computational reasons and to guarantee that our classifiers encounter a high number of training instances, we randomly subsampled 500 tiles per slide at each epoch. Note that if a slide contained <500 tiles that passed the QC checks, all tiles from that slide were used. During inference, the slide-level score is computed with mean pooling leading to the results in Table 1. As we can see, the models trained at 5x (Macro F1 = 0.79) or 10x magnification (Macro F1 = 0.8) obtain comparable results. Looking at the per-category F1 score, we can see that in some critical situations the model suffers from the absence of microscopic information, this is the case for 4 classes that achieve the highest F1 score for 20x magnification: Mesothelioma (MESO), Ovarian serous cystadenocarcinoma (OV), Thyroid carcinoma (THCA), Uterine Carcinosarcoma (UCS).

**Table 1:** Results obtained training the plain ResNet to perform the cancer type classification.

	**Plain ResNet**		
	**5x**	**10x**	**20x**
Weighted F1	0.86	0.85	0.83
Macro F1	0.79	0.80	0.77
ACC	0.43	0.67	0.64
BLCA	0.74	0.78	0.71
BRCA	0.92	0.91	0.90
CESC	0.82	0.75	0.74
CHOL	0.50	0.55	0.25
COAD	0.86	0.86	0.83
DLBC	0.00	0.00	0.00
ESCA	0.74	0.68	0.61
GBM	0.91	0.91	0.90
HNSC	0.88	0.84	0.80
KICH	0.90	0.97	0.90
KIRC	0.93	0.94	0.90
KIRP	0.79	0.75	0.74
LGG	0.95	0.97	0.94
LIHC	0.89	0.87	0.87
LUAD	0.79	0.77	0.78
LUSC	0.75	0.72	0.70
MESO	0.57	0.67	0.70
OV	0.90	0.91	1.00
PAAD	0.92	0.84	0.82
PCPG	0.96	0.96	0.93
PRAD	0.96	0.94	0.95
SARC	0.80	0.80	0.70
SKCM	0.83	0.87	0.83
STAD	0.84	0.80	0.80
TGCT	0.95	0.97	0.92
THCA	0.95	0.93	0.96
THYM	0.94	0.86	0.83
UCEC	0.88	0.87	0.83
UCS	0.37	0.63	0.72

*Rows denote the F1 score for each tumor type individually. The value of the best performing magnification is highlighted in green. The top two rows show the aggregated classification results denoted by the weighted F1 and macro F1 scores for the overall classification across all different tumor types*.

The performances of the models change considerably according to the embeddings used as input. This shows that the algorithms are highly dependent on the quality of the generated embeddings (see Table 2). In particular, MIL performs better by 18 percentage points for Macro F1, when taking as input embeddings generated from the ResNet18 trained on the entire dataset, compared to embeddings from BYOL training. The barcode analysis shows a 3 percentage point improvement when using embeddings from BYOL training. For the experiments below, we considered only the best performing embedding type for each classification method, namely BYOL embeddings for the barcode approach, and plain ResNet18 for MIL approach.

**Table 2:** The embedding is important for the downstream classifiers.

	**Plain ResNet embeddings**	**BYOL ResNet embeddings**
MIL	0.85	0.67
Barcode Analysis	0.65	0.68

*This table denotes the performance of the MIL and barcode method for both the plain ResNet embedding and the BYOL-trained ResNet embedding. For each combination the macro F1 score is provided for the classification across all tumor types. While the plain ResNet embedding works best for the MIL approach, the barcode approach works best with the BYOL-trained embedding*.

Having established a baseline for single-level models, we now assess the importance of combining multiple magnification levels by multi-scale tiles as input to MIL training and the barcode analysis. We train the models on two different datasets (see Methods and Data).

First, we train on the balanced subset of the data to avoid performance issues due to imbalanced datasets. In Table 3, we show the results obtained using the best embeddings and trained on the balanced dataset. As we can see, the multi-scale outperforms the single-scale models and a consistent improvement is observed for multi-scale models. In the classification task at hand, the combination of three magnification levels leads to the best results (MIL: Macro F1 = 0.94, barcode: Macro F1 = 0.73). When training a single-scale model, the choice of a magnification level can be critical. In a multi-scale setting, the combination of information coming from different levels is overall beneficial.

**Table 3:** Results obtained performing MIL and the barcode analysis on single-scale and multi-scale inputs.

	**MIL**					**Barcode**				
	**5x**	**10x**	**20x**	**5x + 10x**	**5x + 10x + 20x**	**5x**	**10x**	**20x**	**5x + 10x**	**5x + 10x + 20x**
Weighted F1	0.88	0.86	0.91	0.93	0.94	0.69	0.58	0.65	0.72	0.74
Macro F1	0.87	0.85	0.88	0.90	0.94	0.67	0.55	0.62	0.70	0.73
MCC	0.88	0.86	0.9	0.93	0.94	0.68	0.57	0.64	0.71	0.74
ACC	0.86	0.86	0.95	0.95	0.95	0.53	0.48	0.58	0.59	0.60
BLCA	0.92	0.90	0.95	0.97	0.98	0.60	0.20	0.45	0.49	0.55
BRCA	0.84	0.86	0.89	0.95	0.84	0.62	0.49	0.59	0.68	0.71
CESC	0.83	0.86	0.88	0.90	0.95	0.62	0.68	0.57	0.71	0.73
CHOL	0.83	0.80	0.71	0.86	0.83	0.33	0.36	0.36	0.36	0.36
COAD	0.84	0.82	0.88	0.95	0.93	0.68	0.45	0.56	0.65	0.76
DLBC	0.50	0.00	0.00	0.00	1.00	0.67	0.00	0.00	0.67	0.67
ESCA	0.95	0.76	0.93	0.95	0.95	0.68	0.26	0.62	0.65	0.67
GBM	0.89	0.90	0.84	0.95	0.87	0.82	0.79	0.71	0.86	0.86
HNSC	0.78	0.89	0.93	0.90	0.90	0.70	0.34	0.65	0.76	0.79
KICH	0.97	0.97	1.00	0.97	0.98	0.90	0.82	0.75	0.87	0.85
KIRC	0.95	0.97	0.90	0.95	0.97	0.87	0.90	0.90	0.92	0.95
KIRP	0.88	0.95	0.92	0.93	1.00	0.72	0.47	0.55	0.78	0.67
LGG	0.92	0.90	0.93	0.90	0.90	0.95	0.90	0.90	0.95	0.95
LIHC	0.92	0.84	0.89	0.92	0.95	0.55	0.58	0.57	0.59	0.63
LUAD	0.79	0.76	0.77	0.85	0.84	0.60	0.58	0.64	0.63	0.70
LUSC	0.72	0.68	0.68	0.73	0.79	0.35	0.28	0.47	0.38	0.49
MESO	0.83	0.85	0.97	1.00	1.00	0.37	0.21	0.21	0.47	0.32
OV	0.90	0.98	1.00	0.98	0.95	0.68	0.59	0.53	0.74	0.78
PAAD	0.90	0.90	0.92	0.95	0.98	0.83	0.51	0.80	0.79	0.82
PCPG	0.97	0.98	1.00	1.00	0.97	0.76	0.68	0.88	0.81	0.90
PRAD	1.00	1.00	1.00	1.00	1.00	0.97	0.87	0.87	0.97	0.97
SARC	0.89	0.90	0.97	0.92	0.95	0.61	0.70	0.58	0.73	0.76
SKCM	0.78	0.92	0.87	0.92	0.92	0.55	0.59	0.60	0.56	0.74
STAD	0.92	0.82	0.76	0.87	0.89	0.57	0.21	0.40	0.53	0.53
TGCT	0.92	0.95	0.98	1.00	1.00	0.86	0.74	0.80	0.89	0.85
THCA	0.98	0.95	1.00	0.98	1.00	0.95	0.88	0.90	0.95	0.95
THYM	0.93	0.90	1.00	0.95	1.00	0.83	0.85	0.87	0.85	0.85
UCEC	0.86	0.78	0.92	0.95	0.91	0.76	0.65	0.67	0.71	0.74
UCS	0.80	0.70	0.83	0.84	0.90	0.25	0.54	0.52	0.60	0.63

*The results are obtained by training the models on a smaller subsample of the dataset (2,727 slides) and testing on a 20% split. The value of the best performing magnification (or combination thereof) is highlighted in green. The top 3 rows show the aggregated classification results: weighted F1, macro F1 and MCC scores for the overall classification across all different tumor types on the test set*.

**Figure 3:**
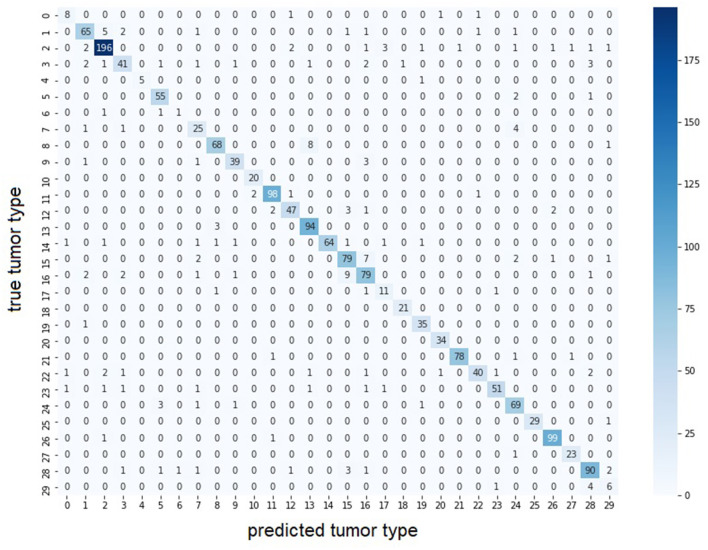
Confusion matrix of multi-scale MIL model trained at 5x and 10x on the entire dataset. The largest values for each row are found on the diagonal indicating the predicted tumor type correspond to the true tumor type. Color intensity represents number of samples in the class. With lower color intensity there are less samples and the task becomes harder. Correspondence between class id and tumor name is presented in Supplementary Table 1.

Second, we use the entire, highly imbalanced dataset. Table 4 illustrates the obtained model performances. We can see that using tiles at 5x and 10x is sufficient to obtain good results (MIL: Macro F1 = 0.87, barcode: Macro F1 = 0.71) and a 0.02 and 0.03 improvements respectively with respect to the best single-scale model is observed. In Figure 3, we report the confusion matrix obtained from the MIL algorithm trained on 5x and 10x tiles. There is a clear predominance of diagonal values, which indicate a good performance of the models. Classes where the model predictions are less accurate (e.g., lower values on the diagonal) correspond to the classes with fewer examples in them. Comparing the macro F1 (MIL: 0.87 barcode: 0.71) and the weighted F1 (MIL: 0.9 barcode: 0.8), the gap between the two scores shows that both the methods and particularly the barcode analysis suffer from the imbalance in the entire dataset.

**Table 4:** Results obtained performing MIL and the barcode analysis on single-scale and multi-scale inputs.

	**MIL**					**Barcode**				
	**5x**	**10x**	**20x**	**5x + 10x**	**5x + 10x + 20x**	**5x**	**10x**	**20x**	**5x + 10x**	**5x + 10x + 20x**
Weighted F1	0.88	0.88	0.87	0.90	0.90	0.78	0.70	0.74	0.80	0.73
Macro F1	0.85	0.84	0.82	0.87	0.86	0.68	0.61	0.64	0.71	0.62
MCC	0.87	0.87	0.86	0.90	0.89	0.77	0.69	0.74	0.80	0.73
ACC	0.86	0.64	0.63	0.86	0.78	0.42	0.47	0.47	0.56	0.43
BLCA	0.83	0.83	0.79	0.86	0.83	0.70	0.48	0.69	0.68	0.65
BRCA	0.93	0.94	0.91	0.93	0.93	0.90	0.82	0.84	0.89	0.82
CESC	0.79	0.77	0.73	0.81	0.80	0.53	0.40	0.53	0.57	0.52
CHOL	0.60	0.80	0.55	0.67	0.83	0.22	0.20	0.00	0.25	0.00
COAD	0.93	0.90	0.85	0.96	0.90	0.82	0.75	0.81	0.86	0.77
DLBC	0.80	0.50	0.00	0.50	0.00	0.00	0.00	0.00	0.00	0.00
ESCA	0.68	0.69	0.73	0.74	0.74	0.50	0.35	0.51	0.55	0.53
GBM	0.85	0.90	0.89	0.90	0.91	0.80	0.76	0.80	0.77	0.80
HNSC	0.87	0.89	0.87	0.88	0.89	0.85	0.51	0.61	0.82	0.62
KICH	0.86	0.87	0.98	0.93	0.92	0.89	0.83	0.80	0.89	0.78
KIRC	0.94	0.95	0.93	0.96	0.95	0.94	0.89	0.89	0.93	0.90
KIRP	0.87	0.82	0.81	0.89	0.90	0.83	0.68	0.68	0.90	0.68
LGG	0.92	0.92	0.94	0.94	0.94	0.89	0.86	0.88	0.84	0.87
LIHC	0.93	0.93	0.91	0.92	0.94	0.82	0.81	0.87	0.87	0.86
LUAD	0.81	0.81	0.85	0.87	0.86	0.69	0.56	0.62	0.72	0.59
LUSC	0.77	0.77	0.79	0.82	0.82	0.60	0.54	0.61	0.66	0.60
MESO	0.67	0.67	0.57	0.73	0.83	0.11	0.11	0.29	0.09	0.20
OV	0.98	0.93	0.93	0.98	1.00	0.67	0.67	0.56	0.70	0.56
PAAD	0.90	0.86	0.89	0.93	0.89	0.72	0.55	0.59	0.75	0.42
PCPG	0.97	0.94	0.96	0.96	0.96	0.90	0.84	0.91	0.94	0.92
PRAD	0.94	0.96	0.96	0.97	0.97	0.96	0.97	0.91	0.97	0.88
SARC	0.84	0.80	0.86	0.85	0.85	0.73	0.63	0.70	0.84	0.70
SKCM	0.85	0.90	0.88	0.93	0.92	0.59	0.53	0.59	0.72	0.60
STAD	0.85	0.88	0.86	0.89	0.86	0.66	0.50	0.66	0.73	0.60
TGCT	0.95	0.93	0.95	0.97	0.97	0.87	0.79	0.75	0.89	0.79
THCA	0.97	0.98	0.97	0.96	0.98	0.93	0.95	0.94	0.94	0.94
THYM	0.93	0.96	0.92	0.96	0.96	0.78	0.78	0.78	0.77	0.79
UCEC	0.87	0.87	0.87	0.91	0.89	0.85	0.77	0.77	0.84	0.77
UCS	0.43	0.52	0.70	0.58	0.64	0.22	0.32	0.12	0.45	0.13

*The results are obtained by training the model on the entire set of slides, after reserving a 20% split for testing. The value of the best performing magnification (or combination thereof) is highlighted in green. The top 3 rows show the aggregated classification results: weighted F1, macro F1 and MCC scores for the overall classification across all different tumor types on the test set*.

## Conclusion and Discussion

In this work, we performed a comparison of single-scale and multi-scale models using several alternatives in terms of tile extraction and models for classification. Our goal was to provide the models with more contextual information by combining different magnification levels. Our results support our hypothesis that multi-scale models outperform the single-scale models as demonstrated by the application of two different training methods, i.e., the MIL and the barcode approaches.

The combination of multiple scales has recently been an interesting topic in DP and different approaches to extract and combine tiles at different magnifications have been proposed for different tasks (22) introduces a naive multiscale models in a MIL setting by pooling the predictions obtained from the single-scale models (23) proposes a multi-scale CNN with a branch for each magnification level to predict tumor mutational burden (7, 8, 24) integrate patches at multiple scales for tumor segmentation (9) presents a multi-scale multi-branch architecture for classification and (10–12) combine MIL and multi-scale inputs. All of these methods show significant improvements of the multi-scale models with respect to the single-scale's.

The main outcome of this paper is to provide an overview of a complete pipeline, with different alternatives, from multi-scale tile extraction to model training using variants of the MIL and the barcode approaches, which can be both directly adapted to deal with multi-scale inputs. Moreover, we follow the recent developments of self-supervision (25–27) and we use domain-specific feature extractors, in a self-supervised and supervised way, as a better alternative to pretraining on ImageNet. As in the previously mentioned references, we show the benefits of combining multiple scales, but also the challenges in terms of cloud computing and data engineering.

The challenge of using multi-scale methods resides in limitations provided by (cloud) computing infrastructures and their costs. As shown in this work, the extraction of concentric tiles is storage intensive. This approach generates a number of tiles equivalent to the number of tiles at the highest magnification level but multiplied by the of magnification levels. Moreover, the tiles of the lower magnification levels overlap significantly with each other. To alleviate this issue, we implemented an alternative version that extracted tiles on the fly rather than storing them on beforehand. Unfortunately, limitations in the number of I/O operations required to construct the concentric tiles rendered this implementation unfeasible to execute in a reasonable time. That is not to say that it is impossible to do, but highlights the importance of data engineering when dealing with these high amounts of data. Apart from these technical considerations, for several applications the use of multi-scale approaches might be beneficial and lead to more insights and valuable results as shown in this study.

Based on our findings we provide some rules of thumb. Firstly, if the slide-level classification task works to the expected accuracy while using a single scale, there is no need to use multiple scales. That said, our results indicate that using multiple-scales generally outperforms single-scales analyses. Therefore, when compute time and storage requirements are not an issue, we recommend using multi-scale approaches for slide-level classification tasks. The next recommendation relates to what magnification levels are best to use. The answer depends on the specific task at hand. When macroscopic structures (such as organ-specific patterns) are required, we found that using a low magnification such as 5x is useful. On the other hand, when microscopic patterns such as the tumor environment are required, we recommend adding 20x data. As seen in the experiments (Tables 3, 4), for tumors in the same tissue (e.g. LUSC and LUAD are both lung cancers), the lack of details in single-scale models penalizes the model as it cannot correctly recognize the type of cancer. In these cases, the combination of different magnification levels might lead to more valuable results. Adding more magnification levels generally improves the performance further but with smaller gains and the decision has to be made between the trade-off of accuracy vs. compute and storage requirements. In future work we would extend our research to investigate the benefits of multi-scale methods for other digital histopathology tasks, such as tissue segmentation, semantic segmentation of cellular objects and cancer growth pattern prediction.

## Data Availability Statement

The datasets presented in this study can be found in online repositories. The names of the repository/repositories and accession number(s) can be found in the article/Supplementary Material.

## Author Contributions

MD'A, PS, and BT-N designed the work and wrote the manuscript. MD'A and PS implemented the models, ran the simulations, and analyzed the data. All authors contributed to the article and approved the submitted version.

## Conflict of Interest

MD'A and BT-N were employed by F. Hoffmann-La Roche AG. PS was employed by Billennium by Order of Roche Polska Sp. z o.o.

## Publisher's Note

All claims expressed in this article are solely those of the authors and do not necessarily represent those of their affiliated organizations, or those of the publisher, the editors and the reviewers. Any product that may be evaluated in this article, or claim that may be made by its manufacturer, is not guaranteed or endorsed by the publisher.

## References

[1] Quellec G Cazuguel G Cochener B Lamard M. Multiple-instance learning for medical image and video analysis. IEEE Rev Biomed Eng. (2017) 10:213–34. 10.1109/RBME.2017.2651164 28092576

[2] Ilse M Tomczak J Welling M. Attention-Based Deep Multiple Instance Learning. In: Proceedings of the 35th International Conference on Machine Learning. PMLR (2018). p. 2127–36. Available online at: https://proceedings.mlr.press/v80/ilse18a.html (accessed March 7, 2022).

[3] Adnan M Kalra S Tizhoosh HR. Representation Learning of Histopathology Images using Graph Neural Networks. ArXiv200407399 Cs Eess (2020). Available online at: http://arxiv.org/abs/2004.07399 (accessed March 4, 2022).

[4] Gueréndel C Arnold P Torben-Nielsen B. Creating small but meaningful representations of digital pathology images. In: Proceedings of the MICCAI Workshop on Computational Pathology. PMLR (2021). p. 206–15. Available online at: https://proceedings.mlr.press/v156/guerendel21a.html (accessed March 7, 2022).

[5] Shin D Kovalenko M Ersoy I Li Y Doll D Shyu CR. PathEdEx - uncovering high-explanatory visual diagnostics heuristics using digital pathology and multiscale gaze data. J Pathol Inform. (2017) 8:29. 10.4103/jpi.jpi_29_17 28828200 PMC5545777

[6] Marini N Otálora S Podareanu D van Rijthoven M van der Laak J Ciompi F. Multi_scale_tools: a python library to exploit multi-scale whole slide images. Front Comput Sci. (2021) 3:68452. 10.3389/fcomp.2021.684521

[7] Schmitz R Madesta F Nielsen M Krause J Steurer S Werner R. Multi-scale fully convolutional neural networks for histopathology image segmentation: from nuclear aberrations to the global tissue architecture. Med Image Anal. (2021) 70:101996. 10.1016/j.media.2021.101996 33647783

[8] van Rijthoven M Balkenhol M Silina K van der Laak J Ciompi F. HookNet: Multi-resolution convolutional neural networks for semantic segmentation in histopathology whole-slide images. Med Image Anal. (2021) 68:101890. 10.1016/j.media.2020.101890 33260110

[9] Kosaraju SC Hao J Koh HM Kang M. Deep-hipo: multi-scale receptive field deep learning for histopathological image analysis. Methods. (2020) 179:3–13. 10.1016/j.ymeth.2020.05.012 32442672

[10] Hashimoto N Fukushima D Koga R Takagi Y Ko K Kohno K. Multi-Scale Domain-Adversarial Multiple-Instance CNN for Cancer Subtype Classification With Unannotated Histopathological Images. (2020). p. 3852–61. Available online at: https://openaccess.thecvf.com/content_CVPR_2020/html/Hashimoto_Multi-scale_Domain-adversarial_Multiple-instance_CNN_for_Cancer_Subtype_Classification_with_Unannotated_CVPR_2020_paper.html 10.1109/CVPR42600.2020.00391 (accessed March 7, 2022).

[11] Marini N Otálora S Ciompi F Silvello G Marchesin S Vatrano S. Multi-scale task multiple instance learning for the classification of digital pathology images with global annotations. In: Proceedings of the MICCAI Workshop on Computational Pathology. PMLR 2021. p. 170–81. Available online at: https://proceedings.mlr.press/v156/marini21a.html (accessed March 7, 2022).

[12] Li B Li Y Eliceiri KW. Dual-Stream Multiple Instance Learning Network for Whole Slide Image Classification with Self-supervised Contrastive Learning. ArXiv201108939 Cs. (2021). Available online at: http://arxiv.org/abs/2011.08939 (accessed March 7, 2022). 10.1109/CVPR46437.2021.01409 PMC8765709 35047230

[13] Weinstein JN Collisson EA Mills GB Shaw KM Ozenberger BA Ellrott K. The cancer genome atlas pan-cancer analysis project. Nat Genet. (2013) 45:1113–20. 10.1038/ng.2764 24071849 PMC3919969

[14] Grill JB Strub F Altché F Tallec C Richemond PH Buchatskaya E. Bootstrap Your Own Latent: A New Approach to Self-Supervised Learning. ArXiv200607733 Cs Stat. (2020). Available online at: http://arxiv.org/abs/2006.07733 (accessed March 7, 2022).

[15] Bussola N Marcolini A Maggio V Jurman G Furlanello C. AI Slipping on Tiles: Data Leakage in Digital Pathology. arXiv. (2020). Available online at: http://arxiv.org/abs/1909.06539 (accessed June 2, 2022).

[16] Eigen D Fergus R. Predicting Depth, Surface Normals Semantic Labels with a Common Multi-Scale Convolutional Architecture. ArXiv14114734 Cs. (2015). Available online at: http://arxiv.org/abs/1411.4734 (accessed March 7, 2022).

[17] Goode A Gilbert B Harkes J Jukic D Satyanarayanan M. OpenSlide: A vendor-neutral software foundation for digital pathology. J Pathol Inform. (2013) 4:27. 10.4103/2153-3539.119005 24244884 PMC3815078

[18] He K Zhang X Ren S Sun J. Deep Residual Learning for Image Recognition. ArXiv151203385 Cs. (2015). Available online at: http://arxiv.org/abs/1512.03385 (accessed March 7, 2022).

[19] Chen T Guestrin C. XGBoost: a scalable tree boosting system. In: Proceedings of the 22nd ACM SIGKDD International Conference on Knowledge Discovery and Data Mining. New York, NY: Association for Computing Machinery (2016). p. 785–94.

[20] Jurman G Riccadonna S Furlanello C. A comparison of MCC and CEN error measures in multi-class prediction. PLoS ONE. (2012) 7:e41882. 10.1371/journal.pone.0041882 22905111 PMC3414515

[21] Wei JW Tafe LJ Linnik YA Vaickus LJ Tomita N Hassanpour S. Pathologist-level classification of histologic patterns on resected lung adenocarcinoma slides with deep neural networks. Sci Rep. (2019) 9:3358. 10.1038/s41598-019-40041-7 30833650 PMC6399447

[22] Campanella G Hanna MG Geneslaw L Miraflor A Werneck Krauss Silva V Busam KJ. Clinical-grade computational pathology using weakly supervised deep learning on whole slide images. Nat Med. (2019) 25:1301–9. 10.1038/s41591-019-0508-1 31308507 PMC7418463

[23] Jain MS Massoud TF. Predicting tumour mutational burden from histopathological images using multiscale deep learning. Nat Mach Intell. (2020) 2:356–62. 10.1038/s42256-020-0190-5

[24] Song Y Zhang L Chen S Ni D Lei B Wang T. Accurate Segmentation of Cervical Cytoplasm and Nuclei Based on Multiscale Convolutional Network and Graph Partitioning. IEEE Trans Biomed Eng. (2015) 62:2421–33. 10.1109/TBME.2015.2430895 25966470

[25] Schirris Y Gavves E Nederlof I Horlings HM Teuwen J. DeepSMILE: Self-Supervised Heterogeneity-Aware Multiple Instance Learning for DNA Damage Response Defect Classification Directly From H&E Whole-Slide Images. ArXiv210709405 Cs Eess. (2021). Available online at: http://arxiv.org/abs/2107.09405 (accessed April 15, 2022).

[26] Ciga O Xu T Martel AL. Self Supervised Contrastive Learning for Digital Histopathology. ArXiv201113971 Cs Eess. (2021). Available online at: http://arxiv.org/abs/2011.13971 (accessed April 15, 2022).

[27] Koohbanani NA Unnikrishnan B Khurram SA Krishnaswamy P Rajpoot N. Self-Path: Self-supervision for Classification of Pathology Images with Limited Annotations. ArXiv200805571 Cs Eess. (2020). Available online at: http://arxiv.org/abs/2008.05571 (accessed April 15, 2022). 10.1109/TMI.2021.3056023 33523807

