# Supplementary material for: A Comparison Between Single- and Multi-Scale Approaches for Classification of Histopathology Images
Source: Front Public Health. 2022 Jul 4;10:892658. doi: 10.3389/fpubh.2022.892658 (PMC9289164; doi:10.3389/fpubh.2022.892658)
Supplement: Supplementary file 1 [file Table_1.pdf]

Supplementary Material

1 Supplementary Tables

Supplementary Table 1. Cancer types with their corresponding acronyms.

Id	Acronym	Cancer type
0	ACC	Adrenocortical carcinoma
1	BLCA	Bladder Urothelial Carcinoma
2	BRCA	Breast invasive carcinoma
3	CESC	Cervical squamous cell carcinoma and endocervical adenocarcinoma
4	CHOL	Cholangiocarcinoma
5	COAD	Colon adenocarcinoma
6	DLBC	Lymphoid Neoplasm Diffuse Large B-cell Lymphoma
7	ESCA	Esophageal carcinoma
8	GBM	Glioblastoma multiforme
9	HNSC	Head and Neck squamous cell carcinoma
10	KICH	Kidney Chromophobe
11	KIRC	Kidney renal clear cell carcinoma
12	KIRP	Kidney renal papillary cell carcinoma
13	LGG	Brain Lower Grade Glioma
14	LIHC	Liver hepatocellular carcinoma
15	LUAD	Lung adenocarcinoma
16	LUSC	Lung squamous cell carcinoma
17	MESO	Mesothelioma

18	OV	Ovarian serous cystadenocarcinoma
19	PAAD	Pancreatic adenocarcinoma
20	PCPG	Pheochromocytoma and Paraganglioma
21	PRAD	Prostate adenocarcinoma
22	SARC	Sarcoma
23	SKCM	Skin Cutaneous Melanoma
24	STAD	Stomach adenocarcinoma
25	TGCT	Testicular Germ Cell Tumors
26	THCA	Thyroid carcinoma
27	THYM	Thymoma
28	UCEC	Uterine Corpus Endometrial Carcinoma
29	UCS	Uterine Carcinosarcoma

Supplementary Table 2. Overview of the TCGA dataset.

Acronym	Train+validation sets slides	Test set slides
BRCA	842	211
KIRC	410	102
THCA	405	101
UCEC	403	101
LGG	390	97
LUSC	382	95
LUAD	374	93
COAD	341	85
PRAD	322	81
GBM	310	78

BLCA	309	77
STAD	300	75
LIHC	288	72
SKCM	232	58
KIRP	220	55
CESC	214	54
SARC	199	50
HNSC	175	44
PAAD	142	36
PCPG	136	34
ESCA	125	31
TGCT	119	30
THYM	97	24
OV	85	21
KICH	81	20
MESO	60	15
ACC	45	11
UCS	42	11
CHOL	29	7
DLBC	10	3

Supplementary Table 3. Validation set results for 5 fold Monte Carlo cross-validation with 95% confidence interval for barcode approach.

	**Full Dataset**					**Subsampled Dataset**				
	**5x**	**10x**	**20x**	**5x + 10x**	**5x + 10x + 20x**	**5x**	**10x**	**20x**	**5x + 10x**	**5x + 10x + 20x**
Weighted F1	0.79 (0.76, 0.81)	0.70 (0.68, 0.73)	0.74 (0.71, 0.77)	0.81 (0.79, 0.83)	0.72 (0.69, 0.75)	0.71 (0.67, 0.75)	0.58 (0.56, 0.61)	0.66 (0.62, 0.70)	0.73 (0.70, 0.77)	0.76 (0.73, 0.79)
Macro F1	0.70 (0.68, 0.72)	0.62 (0.59, 0.65)	0.65 (0.62, 0.69)	0.73 (0.71, 0.76)	0.63 (0.59, 0.67)	0.69 (0.65, 0.74)	0.57 (0.53, 0.60)	0.63 (0.58, 0.69)	0.71 (0.67, 0.76)	0.74 (0.70, 0.79)
